# Erosion Failure of a Soil Slope by Heavy Rain: Laboratory Investigation and Modified GA Model of Soil Slope Failure

**DOI:** 10.3390/ijerph16061075

**Published:** 2019-03-26

**Authors:** Xiaofei Jing, Yulong Chen, Changshu Pan, Tianwei Yin, Wensong Wang, Xiaohua Fan

**Affiliations:** 1 School of Safety Engineering, Chongqing University of Science and Technology, Chongqing 401331, China; xfjing@cqust.edu.cn (X.J.); fxh08@163.com (X.F.); 2 School of Civil Engineering, University of Queensland, Brisbane QLD 4072, Australia; t.yin@uq.net.au; 3 Department of Hydraulic Engineering, Tsinghua University, Beijing 100084, China; 4 Chongqing GaoXin engineering Survey and Design Institute Ltd., Co., Chongqing 401121, China; pcsqsl@163.com; 5 College of Resources and Environmental Sciences, Chongqing University, Chongqing 400030, China; sdgxwws@163.com

**Keywords:** soil slope, rainfall erosion, wetting front, slope angle, model test

## Abstract

Rainfall has been identified as one of the main causes for slope failures in areas where high annual rainfall is experienced. The slope angle is important for its stability during rainfall. This paper aimed to determine the impact of the angle of soil slope on the migration of wetting front in rainfall. The results proved that under the same rainfall condition, more runoff was generated with the increase of slope angle, which resulted in more serious erosion of the soil and the ascent of wetting front. A modified Green-Ampt (GA) model of wetting front was also proposed considering the seepage in the saturated zone and the slope angle. These findings will provide insights into the rainfall-induced failure of soil slopes in terms of angle.

## 1. Introduction

Rainfall is recognized as the major cause of slope instability [1]. A large number of rainfall-induced landslides have happened in recent years [2,3,4]. For example, on December 15, 2014, landslides in Indonesia caused by continuous rainstorms resulted in 56 deaths and 52 missing persons; landslides in northeastern Nepal on June 10, 2015, resulted in 36 deaths and dozens of missing persons; landslides in northwestern Colombia on May 18, 2015, resulted in 83 deaths, dozens of injuries, and more than 700 homeless people. These landslide accidents are all caused by rainfall. Therefore, it is necessary to have an in-depth understanding of the slope failure modes triggered by rainfall in order to prevent landslides.

The infiltration of rainfall into soil and its effect on slope failure has been of particular interest in the past few years [5,6,7,8,9,10,11,12,13,14]. Rahardjo et al. [15] performed a series of parametric studies to understand the significance of hydrological and geotechnical parameters of a slope on its rainfall-induced instability. They revealed that rainfall intensity, soil properties, location of the ground water table, and the slope geometry (angle, height) play a significant role in the rain-induced instability of a slope. Wang et al. [16] found that the function of shear stress and yield stress of slopes in rainfall is related to the angle. The angle of the slope is a key geometrical parameter that controls the extent of the area covered by the slope and its stability. In developed/developing areas, there is a great demand to reduce the area covered by the steep slopes.

Unsaturated soil slopes can be stable with a very steep slope angle until it is exposed to rainfall [17,18], and its stability gradually decreases or is even destroyed under the action of rainfall [19,20,21]. The mechanism of slope failure induced by rainfall can vary depending on the slope angle. It is important to understand the mechanism and initiation of failure of a slope to provide better countermeasures against the failure. This paper presents the results of the slope model tests conducted to investigate the effects of slope angle on the erosion of soil slope and the migration of the wetting front in rainfall. The analysis of wetting front and rainfall erosion of soil slopes with different slope angles was achieved using a self-designed slope rainfall failure test device (SRFTD), which consisted of a glass flume, an observation system for the wetting front, and a rainfall system. In addition, a modified GA model that considers the factor of slope angles was proposed based on the original model. This model could be used for predicting the position of the wetting front and estimating the impact of rainfall erosion on a slope, which will help to provide information for the risk assessment of a soil slope.

## 2. Experimental Test

### 2.1. Experimental Materials and Facilities

The soil slope was made of clay soil whose basic properties are presented in Table 1. According to the weather conditions recorded by Chongqing Meteorological Bureau (in China) in the past 10 years, the ideal intensity of rainfall was set to 1.7 mm/min. Each slope has experienced four rounds of shower (15 min for one round), and there needed to be a 15-min gap between each round, so the overall test lasted for 135 min. During the test, rainwater fell into the flume and flowed out through the soil slope. Conditions of the rainfall and the dimensions of the soil slope are shown in Table 2.

**Figure 1:**
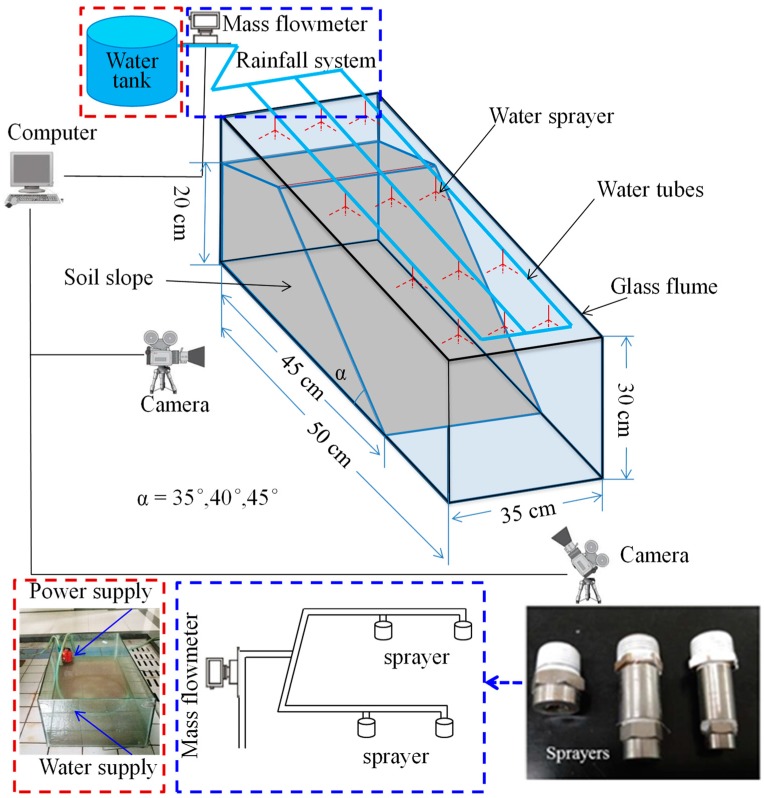
The schematic diagram of slope rainfall failure test device (SRFTD).

The experimental facilities (SRFTD) consisted of a glass flume, an observation system for the wetting front, and an artificial rainfall simulator (Figure 1). The glass flume had an internal dimension of 50 cm (length) by 35 cm (width) by 30 cm (height). The inner wall of the flume was lubricated by Vaseline in order to reduce the friction. The process of erosion and the change of the wetting front could be monitored using a high-resolution camera (Camera model: Sony/HDR-CX680, Camera resolution: 1920 × 1080/50p, Sony (China) Co., Ltd., Beijing, China). Grid lines were drawn on one side of the flume in order to measure the deformation and wetting front of the slope. Four sprayers were installed on the top of the model tank to simulate the rainfall. In accordance with the slope dimension of 45 cm (length) by 35 cm (width) by 20 cm (height), slopes with angles of 35°, 40°, and 45° were tested in this study.

### 2.2. Experiment Results and Discussion

#### 2.2.1. Rainfall Infiltration

**Figure 2:**
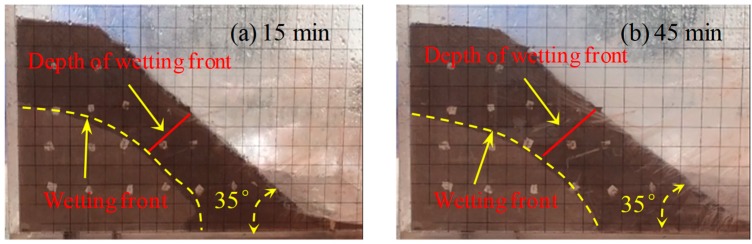

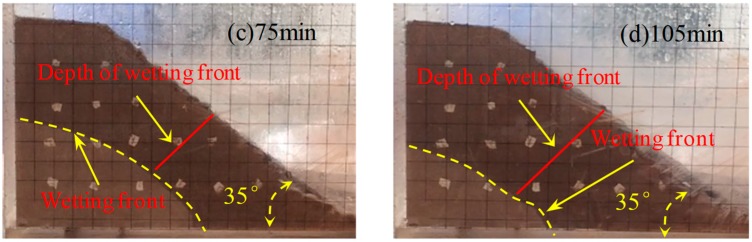
The characteristics of the 35° slope wetting front in rainfall conditions: (**a**) 15 min, (**b**) 45 min, (**c**) 75 min, and (**d**) 105 min.

**Figure 3:**
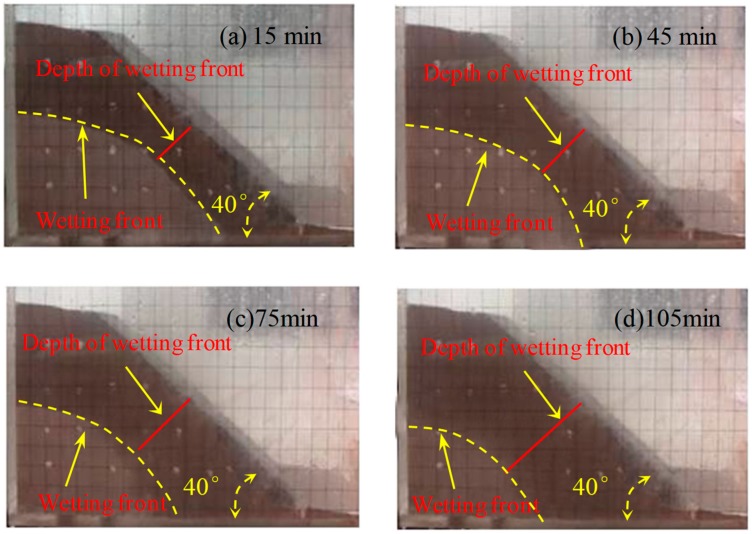
The characteristics of the 40° slope wetting front in rainfall conditions: (**a**) 15 min, (**b**) 45 min, (**c**) 75 min, and (**d**) 105 min.

**Figure 4:**
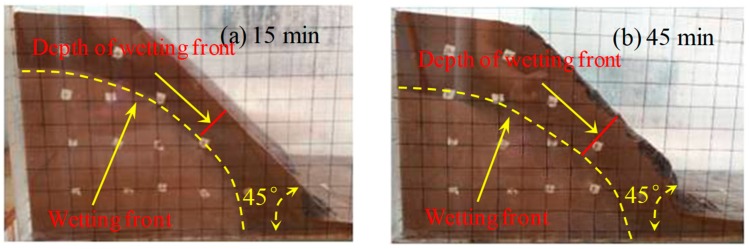

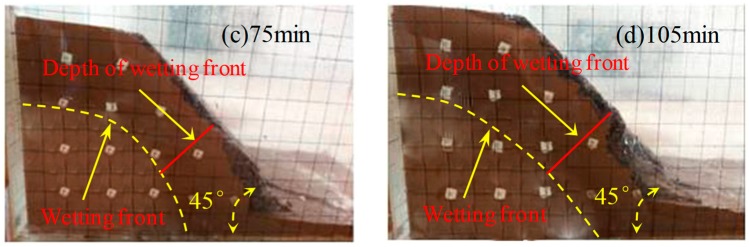
The characteristics of the 45° slope wetting front in rainfall conditions: (**a**) 15 min, (**b**) 45 min, (**c**) 75 min, and (**d**) 105 min.

**Figure 5:**
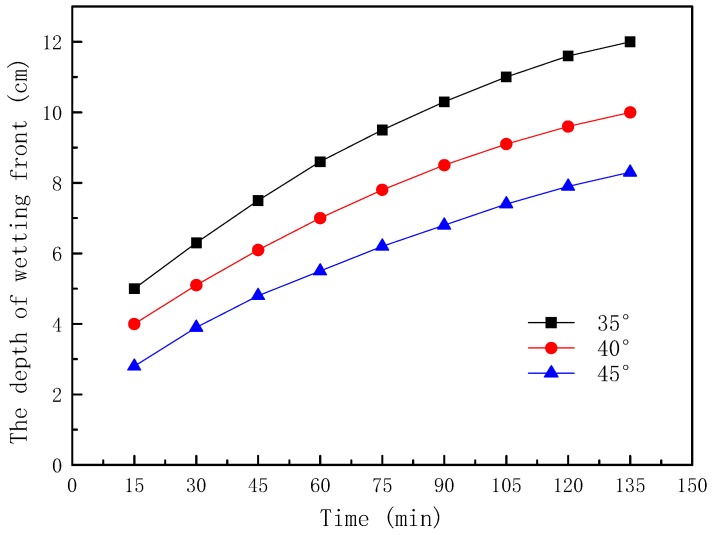
The depth of the wetting front with different slopes.

The wetting fronts of the 35°, 40°, and 45° slopes are showed in Figure 2, Figure 3 and Figure 4, respectively. The depth of the wetting front is the length of the wet part perpendicular to the slope at the middle of the slope. Figure 5 illustrates the variation in the depth of the wetting front over the duration of rainfall for each tested slope.

As can be seen from Figure 2, Figure 3 and Figure 4, the rate of rain infiltration has changed from fast to slow. The main reason was that the soil permeability was high, and rainwater completely infiltrated into the soil, making the soil gradually saturated in the initial stage; when the surface soil was saturated, rainwater partly infiltrated into the soil and formed part of the runoff. It can also be found that the steeper the slope, the greater the loss of soil. The reason was that when the soil was softened by the rainwater, the molecular force between particles gradually disappeared and the particles displaced. At the same time, the infiltration of rainwater increased the bulk density of the soil. Furthermore, the larger the slope angle, the more obvious the gravity effect of the soil was, and the greater the tendency of the soil to slide [22].

It can be seen from Figure 5 that the depth of the wetting front increased with the increase of the slope angle, which indicated that the slope angle affected the infiltration process of the rainwater. The main reason was that the steeper the slope was, the shorter the time the rainwater stayed on the slope, and a larger amount of rainwater ran away in the form of runoff, with only a small amount of rainwater infiltrating into the soil.

#### 2.2.2. Runoff and Soil Erosion

**Figure 6:**
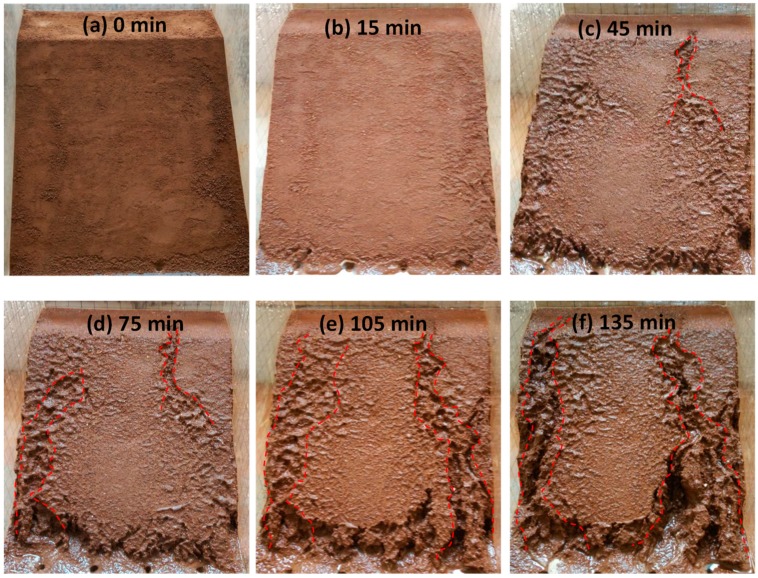
The erosion of the 35° slope surface: (**a**) 0 min, (**b**) 15 min, (**c**) 45 min, (**d**) 75 min, (**e**) 105 min, and (**f**) 135 min.

**Figure 7:**
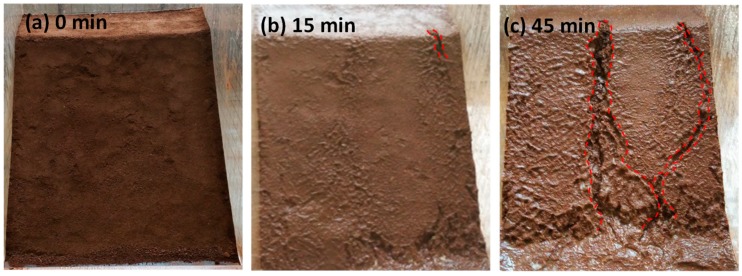

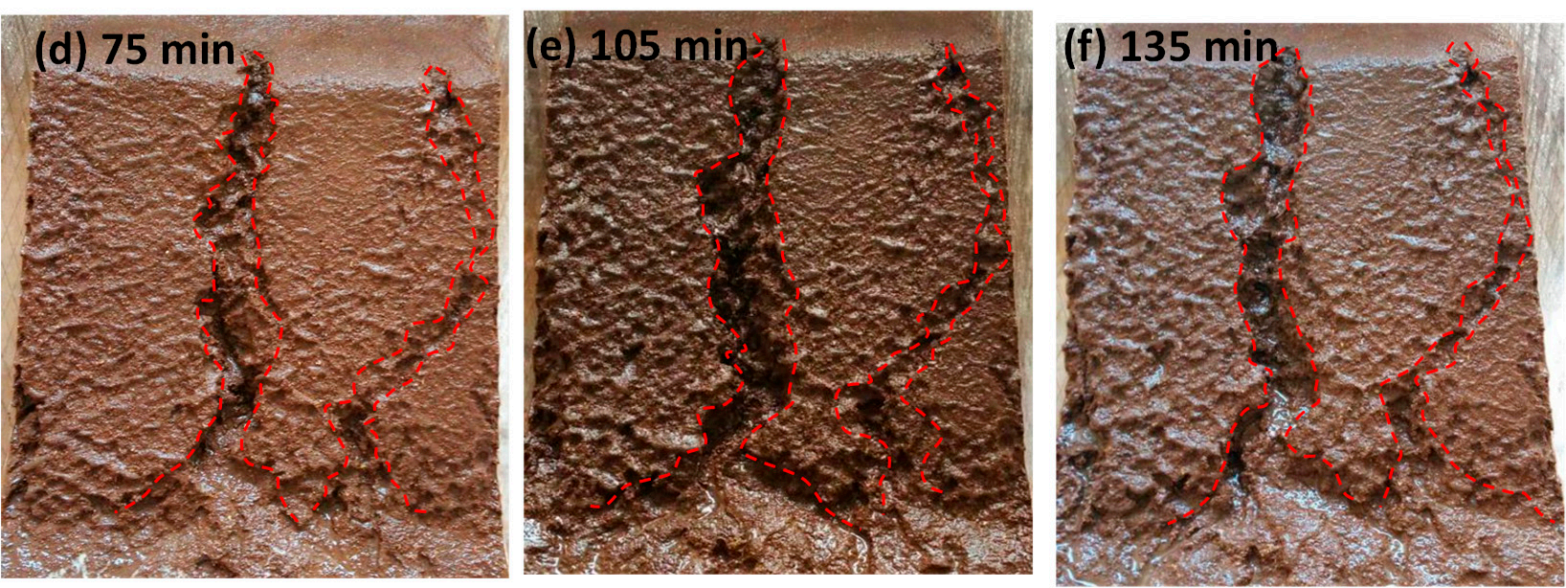
The erosion of the 40° slope surface: (**a**) 0 min, (**b**) 15 min, (**c**) 45 min, (**d**) 75 min, (**e**) 105 min, and (**f**) 135 min.

**Figure 8:**
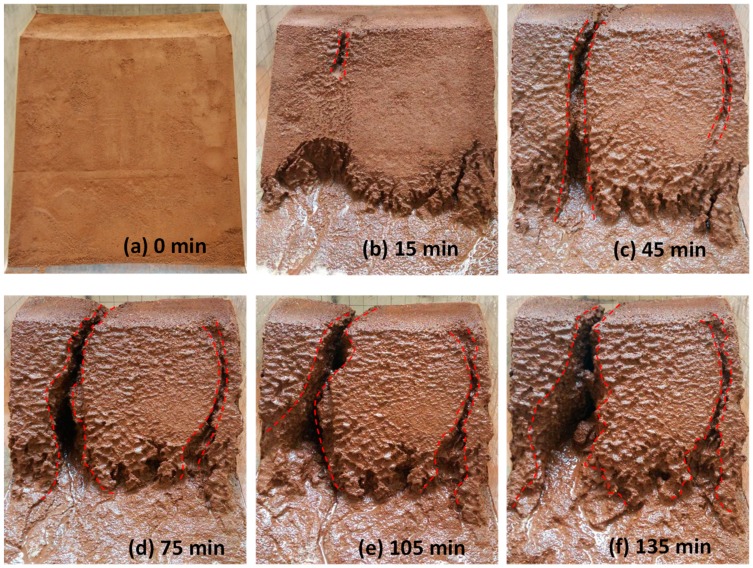
The erosion of the 45° slope surface: (**a**) 0 min, (**b**) 15 min, (**c**) 45 min, (**d**) 75 min, (**e**) 105 min, and (**f**) 135 min.

The process of surface erosion is shown in Figure 6, Figure 7, Figure 8. It was observed that the soil was eroded by the surface runoff formed on the slope. Several channels were formed as the rainwater flushed the soil, which eventually grew to become gullies. With the slope angle increasing, the erosion caused by surface runoff became more serious.

For the slope with an angle of 35°, the effect of surface runoff on soil erosion was not significant over the duration of the first round. Only slight erosion occurred at the bottom of the slope (Figure 6b). As the rainfall time increased, more soil was washed away, leading to the appearance of several narrow gullies. These gullies were gradually getting larger due to erosion (Figure 6c–e). At the end of the tests, the largest width of the gully was found to be 5.0 cm, and the erosion on the bottom of the soil slope appeared to be more serious (Figure 6f).

The soil erosion rate of the 40° slope was faster than that of the 35° slope, resulting in wider gullies. At the end of the test, the largest width of gully was found to be 6.0 cm (Figure 7).

For the slope with an angle of 45°, the width of the gully had increased to 9.0 cm at the end of the test. It was observed that the soil on the two sides of the gully had flushed away. The gully widened all the way down to the bottom, and eventually appeared in the shape of a horn (Figure 8).

As shown in Figure 6, Figure 7 and Figure 8, the erosion process of rainwater on the slope is as follows: (1) Non-destructive stage: runoff flows directly from the slope without causing obvious damage to the slope; the reason was that the softening effect of rainwater on the slope soil has not yet started. (2) Channel formation stage: channels gradually formed on the slope with the continuous rainfall; the reason was that the scouring effect of runoff and the softening effect of seepage water were more obvious with the increase of time. (3) Gully formation stage: under the continuous action of slope runoff and seepage water, the gully gradually widened and deepened, and finally formed a gully.

In the experiment, not only the seepage on the soil surface was found, but also the rainfall runoff on the slope was found. On the one hand, runoff eroded the surface of the slope, causing soil erosion and slope damage; on the other hand, runoff may have reduced the flow of soil infiltration along the slope, thereby reducing the infiltration rate. Therefore, the rainwater falling to the slope can be divided into the seepage part *q1* and the runoff part *q2* [23]. When the rainfall intensity is smaller than the soil permeability, *q1* ≠ 0 and *q2* = 0. At this time, the slope is only affected by seepage water. When rainfall is stronger than soil permeability, *q1* ≠ 0, *q2* ≠ 0. At this time, the slope is not only subjected to a seepage force, but also to erosion via runoff.

## 3. Rainfall Infiltration Model of the Soil Slope

The traditional Green-Ampt model is shown in Equation (1) [22,24]. The model expresses the relationship between infiltration rate and saturated permeability coefficient, surface water head, average matrix suction head at wetting front, and generalized depth of wetting front (vertical downward depth).

(1) $$f=k_{s}\left(\frac{Z_{w}+h_{0}+S_{f}}{Z_{w}}\right)$$

where *f* is the infiltration rate (cm/min), *k_s_* is the saturated permeability coefficient of soil, *S_f_* is the matrix absorption water head in the wetting front, *Z_w_* is the vertical downward depth (cm) of the wetting front, and *h_0_* is the total water potential at the surface.

Meanwhile, in the GA model, the cumulative infiltration *F_w_* of rainwater is as follows:

(2) $$F_{w}=\left(\theta_{s}-\theta_{i}\right)Z_{w}$$

where *θ_s_* and *θ_i_* are the saturation moisture content and initial moisture content, respectively, and *F_w_* is the cumulative infiltration.

Infiltration rate is the time derivative of the infiltration quantity, and the relationship between the depth of wetting front and time can be obtained by combining Equation (1):

(3) $$t=\frac{\left(\theta_{s}-\theta_{i}\right)}{k_{s}}\left[Z_{w}-\left(h_{0}+S_{f}\right)ln\frac{Z_{w}+S_{f}+h_{0}}{S_{f}+h_{0}}\right]$$

The traditional GA model assumes that the unsaturated layer is the saturated layer to calculate the cumulative infiltration and does not consider the influence of the slope gradient on infiltration, which is unreasonable.

When calculating the cumulative infiltration, the infiltration of the saturated zone and unsaturated zone should be calculated separately. Then, the actual cumulative infiltration of rainwater can be obtained by summing up the cumulative infiltration of the two parts. At this time, the equations of cumulative infiltration and infiltration rate are as follows [25]:

(4) $$\begin{array}{l} f=K_{s}(\frac{\frac{1}{2}Z_{w}+h_{0}+S_{f}}{\frac{Z_{w}}{2}})\\ F_{w}=\frac{Z_{w}}{2}\left(\theta_{s}-\theta_{i}\right)+\frac{Z_{w}}{8}\pi \left(\theta_{s}-\theta_{i}\right) \end{array}\}$$

Because infiltration rate is the derivative of the infiltration amount with respect to time, the relationship between the depth of wetting front and time can be obtained using Equation (4). The infiltration depth of the wet front at slope is *Z_p_* = *Z_w_* cos*a* (*Z_p_* is the depth of wetting front perpendicular to the slope, and *a* is the slope angle), and the water head at the surface is very small (*h_0_* ≈ 0). Therefore, the relationship between the depth of the wetting front and the time is as follows:

(5) $$t=\frac{\left(4+\pi \right)\left(\theta_{s}-\theta_{i}\right)}{16k_{s}}\left[\frac{2Z_{p}}{cos\alpha }-4S_{f}ln(1+\frac{Z_{p}}{2S_{f}cos\alpha }\right)]$$

**Figure 9:**
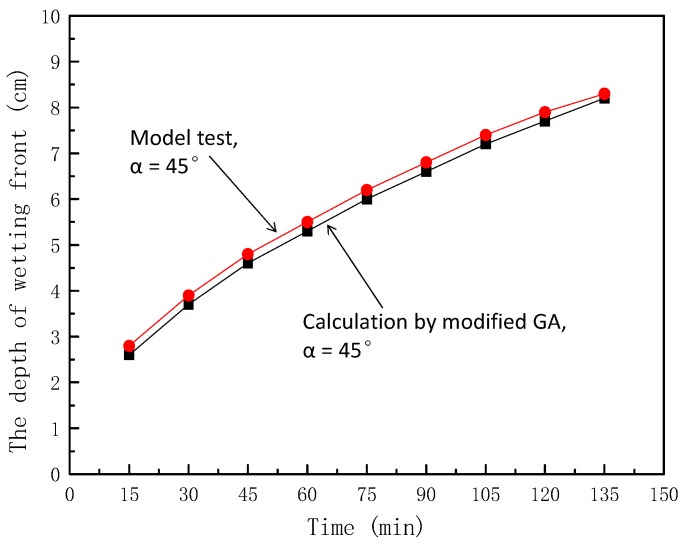
Comparison of calculation and model test values of wetting peak depth (*α* = 45°).

In this experiment, *θ_s_* = 0.42, *θ_i_* = 0.12, *k_s_* = 4 cm/d, and *S_f_* = 20 cm. When *a* = 45°, other parameters were introduced into Equation (5) to obtain the theoretical value of the wetting peak depth perpendicular to the slope, which varied with time, as shown in Figure 9.

From Figure 9, it can be seen that under the condition of a 45° slope, the maximum error was 7.14% and the curve of wetting front depth–time relationship calculated using the modified GA model was in good agreement with the curve of depth–time relationship measured using the model test.

**Figure 10:**
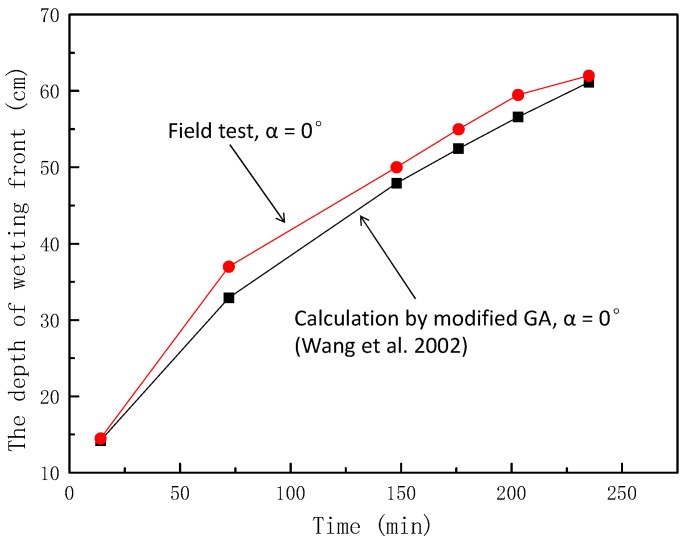
Comparison of calculation and field test values of wetting peak depth (α = 0°).

In order to further verify the reliability of the modified model, the field test data of Wang et al. [26] in the Qinwangchuan area of Gansu Province in China were used for analysis. The parameters of the field test were *θ_s_* = 0.53, *θ_i_* = 0.3, *k_s_* = 0.0048 cm/min, *S_f_* = 150 cm, and *a* = 0°. The theoretical values of the test conditions were obtained using Equation (5), as shown in Figure 10.

From the Figure 10, it can be seen that the maximum error was 11.05%, and the curve of wetting front depth–time of field test were in good agreement with the curve of wetting front depth–time calculated using the modified GA model.

This shows that the modified GA model can feasibly be applied to predict the wetting front depth in the field.

## 4. Conclusions

This paper presents the results of a series of model tests on the behavior of a slope subjected to rainfall with the change of slope angles. The research discussed the failure modes of the slope caused by rainfall, focusing on the change of wetting front and soil erosion of the slope. The following conclusions could be drawn from this study:

(1) Because the slope angle affected the retention time of rainwater on the slope, the slope had an obvious influence on the infiltration process of rainwater. The larger the slope angle, the less seepage water that was transformed by rainwater, the smaller the soil was subjected to a softening effect, and the greater the depth of the wetting front. Moreover, the change rate of the depth of the wetting front de creased with the increase of time.

(2) Slope angle had a significant impact in the scouring process of runoff. The larger the slope, the larger the runoff transformed by rainwater, the greater the erosion effect of runoff on the slope, and the greater the soil loss. Moreover, the scouring process of runoff could be divided into three stages: non-destructive stage, channel formation stage, and gully formation stage.

(3) Based on the traditional GA model, considering the influence of slope and cumulative infiltration in unsaturated zone on seepage flow, a modified GA model was proposed, and it was found that the model was feasible through model test and field test data.

This research provided insights into the rainfall-induced failure for soil slopes in terms of the angle. Further research could investigate grain-size effect on the migration of the wetting front of a soil slope in rainfall.

## Figures and Tables

**Table 1:** Characteristics of the soil sample.

Moisture *ω* (%)	Bulk Density *ρ_s_* (g·cm^−3^)	Void Ratio *e*	Saturation *S_r_*	Liquid Limit *w_L_*	Plastic Limit *W_p_*	Cohesive Force *c* (kPa)	Internal Friction Angle *φ*(°)
12.5	2.73	0.8	0.414	32.8	23.56	23.9	12.51

**Table 2:** Conditions of the rainfall and the dimensions of the soil slope.

Test Number	Slope Dimension (cm)	Slope Angles α (°)	Rainfall (mm/min)
1	45 × 35 × 20	35	1.7
2	45 × 35 × 20	40	1.7
3	45 × 35 × 20	45	1.7
